# Development and validation of reassigned CEA, CYFRA21-1 and NSE-based models for lung cancer diagnosis and prognosis prediction

**DOI:** 10.1186/s12885-022-09728-5

**Published:** 2022-06-22

**Authors:** Jingmin Yuan, Yan Sun, Ke Wang, Zhiyi Wang, Duo Li, Meng Fan, Xiang Bu, Jun Chen, Zhiquan Wu, Hui Geng, Jiamei Wu, Ying Xu, Mingwei Chen, Hui Ren

**Affiliations:** 1 Department of Respiratory and Critical Care Medicine, The First Affiliated Hospital of Xi’an Jiaotong University, 277 Yanta West Road, Xi’an, Shaanxi Province China; 2 Health Science Center, Yangtze University, Jingzhou, China; 3 Shaanxi Health Information Center, Xi’an, China; 4 Medical Department, The First Affiliated Hospital of Xi’an Jiaotong University, Xi’an, China; 5 Physical Examination Center, The First Affiliated Hospital of Xi’an Jiaotong University, Xi’an, China; 6 Shaanxi Huizhong Kangyun Medical Information Co., Ltd., Xi’an, China; 7 Office of Medical Information Management, The First Affiliated Hospital of Xi’an Jiaotong University, Xi’an, China; 8 Shaanxi Provincial Research Center for the Project of Prevention and Treatment of Respiratory Diseases, Xi’an, China; 9 Department of Talent Highland, The First Affiliated Hospital of Xi’an Jiaotong University, Xi’an, China

**Keywords:** Lung cancer, CEA, CYFRA21-1, NSE, Screening, Prognosis

## Abstract

**Background:**

The majority of lung cancer(LC) patients are diagnosed at advanced stage with a poor prognosis. However, there is still no ideal diagnostic and prognostic prediction model for lung cancer.

**Methods:**

Data of CEA, CYFRA21-1 and NSE test of patients with LC and benign lung diseases (BLDs) or healthy people from Physical Examination Center was collected. Samples were divided into three data sets as needed. Reassign three kinds of tumor markers (TMs) according to their distribution characteristics in different populations. Diagnostic and prognostic models were thus established, and independent validation was conducted with other data sets.

**Results:**

The diagnostic prediction model showed good discrimination ability: the area under the receiver operating characteristic curve (AUC) differentiated LC from healthy people and BLDs (diagnosed within 2 months), being 0.88 and 0.84 respectively. Meanwhile, the prognostic prediction model did great in prediction: AUC in training data set and test data set were 0.85 and 0.8 respectively.

**Conclusion:**

Reassigned CEA, CYFRA21-1 and NSE can effectively predict the diagnosis and prognosis of LC. Compared with the same TMs that were considered individually, this diagnostic prediction model can identify high-risk population for LC screening more accurately. The prognostic prediction model could be helpful in making more scientific treatment and follow-up plans for patients.

**Supplementary Information:**

The online version contains supplementary material available at 10.1186/s12885-022-09728-5.

## Introduction

Lung cancer, a malignant tumor with the highest incidence and death rate, causes serious damage to human health [1]. According to GLOBOCAN Estimate, in 2018, newly confirmed cases of LC accounted for 11.6% among all malignancies, and 18.4% among all deaths globally [2]. Poor prognosis is observed in LC patients: their the five-year survival rate in 2019 was only 19.4%, with most of the patients diagnosed at advanced stage [3]. The five-year survival rate can be 90% or higher when the cancer is diagnosed at stage I, but it would be less than 10% when diagnosed at stage IV [4]. Unfortunately, current global rate of LC diagnosis at limited stage is only 16% [5, 6]. Low-dose computed tomography (LDCT) screening scan can reduce LC mortality by 20% [7]. However, LDCT scan has a high false-positive rate [8]. In addition, radiation exposure and unnecessary anxiety also exist [9, 10], for which less than 5% of high-risk population has ever received LDCT screening [11, 12]. Therefore, it is necessary to find an accurate prediction model that identifies high-risk groups of LC.

Serum tumor markers (TMs) such as carcinoembryonic antigen (CEA), cytokeratin-19 fragment (CYFRA21-1) and neuron-specific enolase (NSE) are routinely tested for diagnosis and prognosis of LC for their easy application [13]. TMs values can climb higher than the reference level with aging or certain benign diseases while remain normal in some cancer patients. Since single TM value can only provide limited reference, three kinds of TMs, CEA, CYFRA21-1 and NSE, were selected in this study. Using reassigned TMs of patients who were identified as LC and patients with benign lung diseases (BLDs) or healthy people in physical examination, a LC diagnosis prediction model was built to identify candidates for LDCT screening more accurately. A prognostic model was also established to provide an important basis for making more scientific diagnosis, treatment and follow-up plans for LC patients.

## Methods

### Participants and study design

Data of inpatients who were diagnosed as LC, BLDs with at least two test records, as well as healthy people in Physical Examination Center of The First Affiliated Hospital of Xi’an Jiaotong University from April 2013 to November 2021 was retrospected in this study. All subjects had completed CEA, CYFRA21-1, and NSE tests. Considering that surgery is the standard of care for patients with operable early-stage (stages I or II) [14], in this study, stage I or II of non-small cell lung cancer (NSCLC), and limited stage of small cell lung cancer(SCLC) were collectively referred to as early stage; and the remaining stages are designated advanced stage.

Data was divided into three sets: Data 1, Data 2, and Data 3 (Supplement Fig. 1A). Data 1 consists of cases met high-risk population criteria for lung cancer screening (50 to 75 years old, with a smoking history of at least 20 pack-year), which including 391 patients newly diagnosed as early stage LC (Data 1A) and 772 healthy subjects (Data 1B). Data 2 consists of 68 LC patients (Data 2A) who had TM test records prior to (at least one month) and at the time of diagnosis and 208 BLD patients (Data 2B) who had TM test records at least twice before (with an interval of at least 1 month). Data 3 contains 4,351 LC patients at defined stage (Data 3A), and 2,094 LC patients at undefined stage (Data 3B). There was no overlap of data among these three parts. Written informed consent was waived by The First Affiliated Hospital of Xian Jiaotong University.

**Fig. 1:**
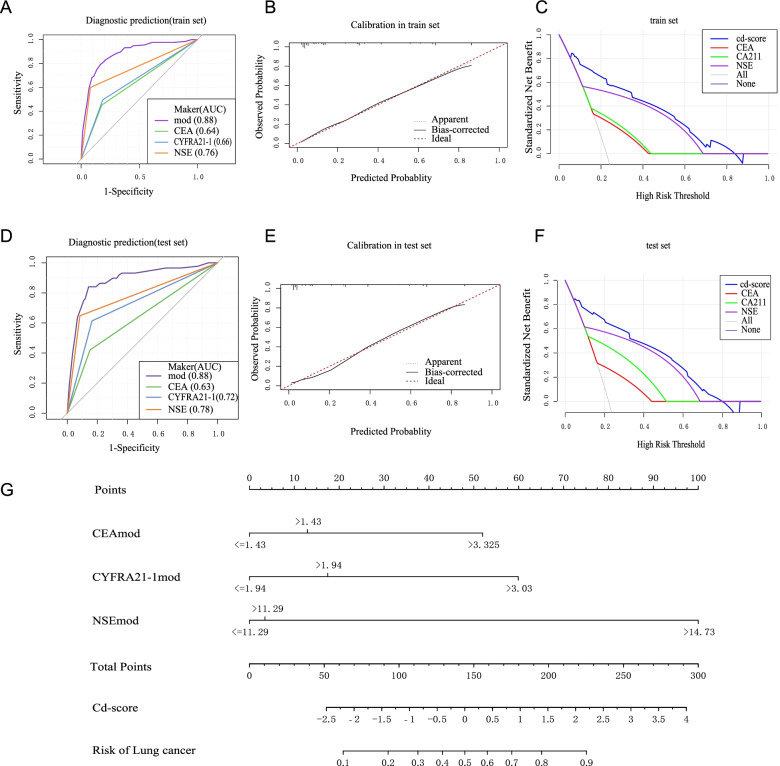
Diagnostic model for LC based on CEA, CYFRA21-1 and NSE. **A** and **D** ROC and corresponding AUCs for diagnostic prediction by cd-score, CEA, CYFRA21-1 and NSE in training data set (**A**) and test data set (**D**). **B** and **E** Calibration curves which show the relationship between the predicted probabilities base on the diagnostic model (cd-score) and actual values of the training data set (**B**) and test data set (**E**). **C** and **F** Decision curve analysis (DCA) of diagnosis based on diagnostic model (cd-score) and each individual TM in training data set (**C**) and test data set (**F**). **G** Nomogram for predicting LC diagnosis based on cd-score. LC, lung cancer. CEA, carcinoembryonic antigen (CEA). CYFRA21-1, cytokeratin-19 fragment. NSE, and neuron-specific enolase

Data 1 was randomly divided into a training set and a test set at a ratio of 7:3. Based on the distribution characteristics of TM in the training data set, TM values were re-assigned (see Supplementary material for more details), and a diagnostic prediction model was then established. We validated the model with the test data set and Data 2 (Supplement Fig. 1B). In order to predict the prognosis, LC patients in Data 3A were divided into a training set and a test data set at a ratio of 7:3. Death was taken as the endpoint event, and survival analysis was carried out with Data 3A’s training data set. A prognostic prediction model was established and validated with Data 3A’s test data set and Data 3B respectively (Supplement Fig. 1C). ROC methods, calibration charts and decision curves were used to evaluate the prediction model. Methods for model establishment and validation were described in section Supplementary material.

### TM measurements

Serum CEA, CYFRA21-1 and NSE were selected in this study. Before initiation of any anticancer treatment, 3 mL peripheral venous blood was extracted into an empty stomach at 4000r/min, and the serum was separated and stored at -80℃ for test. Electrochemiluminescence immunization (Roche Cobas e601) was applied to evaluate three kinds of TMs. The upper limits of reference values are: CEA, 3.4 ng/ mL; CYFRA21-1, 3.3 ng/mL; and NSE, 16.30 ng/mL. In order to reduce the interference of extreme values and ensure the discriminability of the TMs, we re-assigned each TM to be a three-categorical variable when establishing the diagnostic prediction model, and re-assigned each TM as a dichotomous variable when establishing the prognostic prediction model. See Supplementary material for specific assignment methods.

### Statistical analysis

The diagnostic prediction model was established with logistic regression, and comprehensive diagnostic prediction score (cd-score) was thus calculated. Conduct Wilcoxon test to compare the difference in TMs between different populations. Independent sample T test was carried to differentiate two cd-score groups in Data 2, and the area under the receiver operating characteristic curve (AUC) was applied to evaluate the model’s accuracy. The prognostic prediction model was established with COX regression, and the comprehensive prognostic prediction scores (cp-score) were calculated based on the three kinds of TMs. Kaplan–Meier curves and log-rank tests were used to analyze the incidence risk of LC for patients with BLDs and the mortality risk for patients with LC respectively. Time-dependent ROC [15] curve was used to evaluate the model’s predictive ability in the identification of endpoint events occurrence. The agreement between predicted probability and actual outcome was tested with calibration plotting. Finally, evaluated the clinical value of predictive models by decision curve analysis (DCA). *p* < 0.05 was taken as statistically significant. All statistical tests were two-sided and statistical analysis was performed using R version 4.0.2.

## Results

### Characterization of participants

Three kinds of participants were included in this study. Data 1 consists of patients whose age and smoking history meet high-risk population criteria for LC screening. Data 1 was further divided into two parts: Data 1A contains patients at early LC stage, with 391 patients in total (223 males); Data 1B includes all healthy people tested in Physical Examination Center, which is 772 samples. In Data 2, Data 2A comprises 68 LC patients who had TM test within one month before diagnosis, and Data 2B includes 208 BLD patients who had TM test records at least twice (with an interval of one month at least). In Data 3, Data 3A consists 4,351 patients with LC at defined stage, Data 3B includes 2,094 LC patients at undefined stage. Clinical and TM characteristics can be checked in Supplement Table 1.

### Heterogeneity of the three TM values in different groups

The proportion of each TM value greater than the reference one in different groups was calculated. Results showed that TM values of 50% patients with early LC and 25% patients with advanced LC were below the reference (Supplement Fig. 2, Supplement Table 2). This suggests that the discrimination ability of the reference value is quite limited, which is similar to the conclusion of other studies [16]. Afterwards, we made a logarithmic transformation of CEA, CYFRA21-1 and NSE, and described their distribution in different groups. It was found that in healthy people, early LC, and advanced LC patients, the level of three TMs all gradually increased, and the difference among groups showed statistical significance (Supplement Fig. 3A, B&C). For LC patients in different treatment stages, TM level lowered after surgery, and increased significantly after relapse or before death. Through descriptive analysis, it was found that CEA was significantly elevated in lung adenocarcinoma compared with other histological types. Similarly, CYFRA21-1 and NSE were significantly elevated in patients with squamous cell carcinoma and small cell LC, respectively. To sum up, these three TMs are heterogeneous in different groups, for which they can be used to predict the diagnosis and prognosis of LC.

**Fig. 2:**
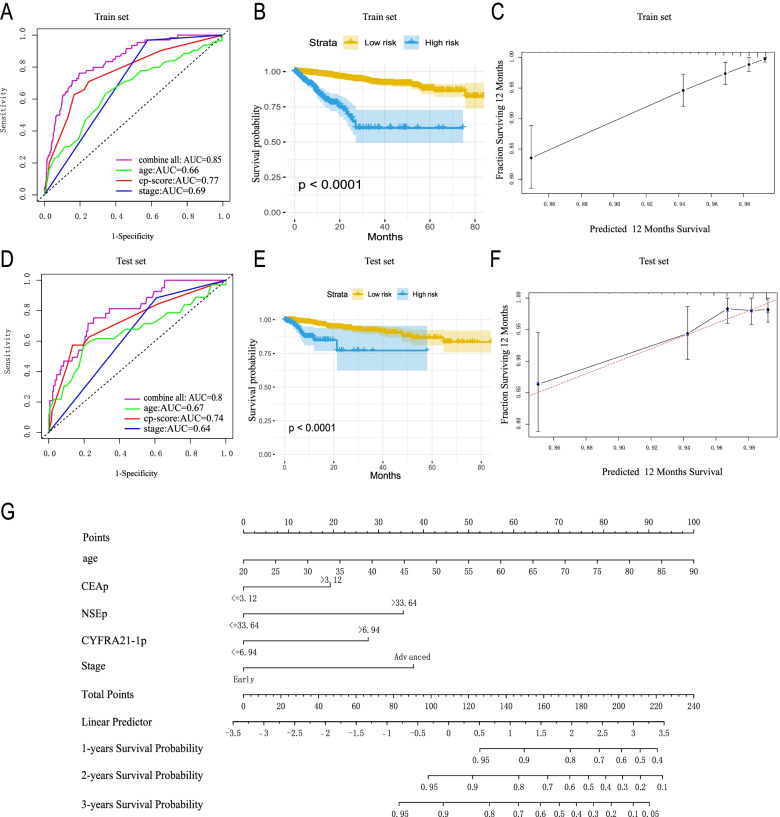
Prognostic model for LC based on cp-score, age and stage. **A** and **D** ROC and corresponding AUCs for 12-month survival predicted by cp-score, age, stage and all combined in the training (A) and test (D) set. **B** and **E** Overall survival curves of patients with LC (both low and high risk of death) according to the combined prognosis score in the training set (B) and test set (E). **C** and **F** Calibration plot of nomogram for predicting 1-year overall survival in the training set (C) and test set (F). (**G**) Nomogram for predicting 1-, 2-, and 3-year overall survival of LC patients using TMs and other clinical factors. LC, lung cancer. TM, tumor marker. CEA, carcinoembryonic antigen. CYFRA21-1, cytokeratin-19 fragment. NSE, and neuron-specific enolase

### TM-based diagnostic prediction model for LC

Data 1 was randomly divided into a training set and a test set at a ratio of 7:3. Based on the training set, re-assigned TMs were used to establish a diagnostic prediction model, which uses logistic regression to obtain the predictive score (cd-score). Results showed that cd-score manifested better prediction ability of LC identification than single TM. AUC of cd-score was 0.88 in both training set and test set, higher than that of single TM, which was 0.63 to 0.78. The difference was statistically significant (Fig. 1A&D). According to Youden-index, calculation suggested that the best threshold of cd-score for diagnosis of LC was -0.88, and corresponding sensitivity and specificity were 0.79 and 0.80, respectively.

The calibration curve revealed that predicted probability and validated probability of the model have high consistency in the training and test set, (Fig. 1B&E). To evaluate the net benefit of cd-score for clinical diagnosis, DCA curves were drawn with the same validation data sets, which showed better effects compared with the control models, suggesting its clinical effectiveness (Fig. 1C&F). The diagnostic prediction model was presented in the form of nomogram (Fig. 1G).

In order to explore the prediction ability of cd-score in surveillance, we analyzed patient’s TM levels before LC diagnosis, and calculated the predictive scores (cd-score 1 and cd-score 2) of patients’ two TM tests in Data 2. For LC patients (Data 2A), cd-score 1 was the score before LC diagnosis and cd-score 2 was the score at diagnosis. The difference between these two scores was statistically significant, and that of the LC group was higher (Supplement Fig. 4A). This suggested that rising of cd-score is in line with the increase of LC risk. Time-dependent ROC curve showed that the AUC of cd-score 1 to discriminate LC diagnosed within 2 months was 0.84, showing a certain predictive ability after TM test (Supplement Fig. 4B), evidencing its role of guidance on the follow-up plan for patients. According to Youden-index, the best threshold was -0.90, based on which Data 2 was divided into a low-risk group and a moderate-risk group. A survival curve was drawn, with LC diagnosis as the endpoint event. Results revealed that this survival curve can distinguish different outcomes of patients in Data 2 (Supplement Fig. 4C).

### TM-based prognostic prediction model for LC

Data 3A (staged LC patients) was randomly divided into a training set and a test set. In the training set, three TMs were re-assigned as binary variables according to their prognostic prediction ability, namely CEAp, CYFRA21-1p and NSEp (see Supplementary material for details) respectively. Included three TMs in COX regression model to generate a TM-based comprehensive prognostic prediction score (cp-score). In the training data set, test data set and Data 3A, time-dependent ROC curves showed that compared with stage, cp-score had higher AUC in 6, 12 and 24 months after the diagnosis, suggesting better distinguishing ability (Supplement Fig. 5). The survival curve also revealed consistent results (Supplement Fig. 6).

To provide better guidance on clinical practice, in the training set, we included CEAp, CYFRA21-1p, NSEp, age and stage into the COX regression model to establish a comprehensive prognosis prediction model (Supplement Table 3), and the test data set was applied for validation. From the time-dependent ROC curve 12 months after the diagnosis, it was found that although cp-score had good predictive ability, the comprehensive model performed better. Its AUC in the training set and test set was 0.85 and 0.8, respectively (Fig. 2 A, D). According to Youden-index, the best threshold was 0.92, which was used to divide the patients into high-risk and low-risk groups. Then the survival curves were drawn, in which results showed a better discrimination effect (Fig. 2 B, E). Through the calibration chart 12 months after the diagnosis, the predicted survival probability obtained from the model was in good agreement with the actual value (Fig. 2C, F). At last, the prognostic model was presented with a nomogram to predict 1, 2 and 3-year survival rates (Fig. 2G).

## Discussion

Current diagnosis rate of LC at early stage is still unsatisfactory. Although LDCT can detect early curable LC and reduce the mortality rate by 20% [7, 17], its high false positive rate affect patients to a certain extent. CEA, CYFRA21-1 and NSE are routinely examined for the diagnosis and prognosis of LC. In this study, model’s diagnosis and prognosis prediction of LC were established and validated.

We reassigned CEA, CYFRA21-1 and NSE based on their distribution in different groups, as TM levels of some people without LC exceed the reference value while that of some LC patients are lower than the reference value [18–20]. Tumor marker levels may be elevated in non-cancer populations by certain factors (such as smoking history, aging, inflammation, etc.). Bjerner et al. found that aging of subjects and active smoking were significant factors associated with high concentrations of CEA, while active smoking was associated with lower concentrations of NSE [20]. In the study of Hao et, al., increased serum CEA levels were associated with aging and some noncancer diseases like lung fibrosis and chronic obstructive pulmonary disease, etc [21]. Barouchos et, al. suggested that TMs were positively associated with inflammatory biomarkers, such as WBC, CRP, ESR etc [22]. In conclusion, confounding factors hve to be considered in lung cancer risk calculation. Samples included in the establishment of the diagnostic prediction model all meet the high-risk population standards for LC screening, making this model applicable for real scenarios.

The comprehensive consideration of TMs in screening is necessary because the elevation of tumor markers is heterogeneous in different histological types of LC. The results of Molina et al. showed that the sensitivity of each individual TM for LC diagnosis was limited: the sensitivity of CEA, CYFRA21-1 and NSE were 56.5%, 56.1% and 19.1%, respectively. It is well known that evaluation of a group of TMs rather than a single one can improve diagnostic performance [23].

In order to explore the ability of cd-score in follow-up, we analyzed patient’s TM test before and at the diagnosis of LC. It was found that the difference between these two tests may provide some valuable information in diagnosis prediction. Results are in line with Molina’s study that abnormal TMs often restore to normal with repeat assessment in people without cancer [23]. After examination, when a subject is followed up for the second time, changes of cd-scores can be also considered. For value increase, follow-up frequency will be increased, or LDCT screening or a diagnostic workup can be carried out directly.

It was showed that these three TMs can also help evaluate the treatment effect and prognosis. TM level after LC surgery was lower than that before, and that would rise again in LC recurrence and before. We included three TMs, age and LC stage into the prognostic prediction model, which is presented as a more intuitive nomogram that predicts the survival rate of LC patients in 1, 2, and 3 years after diagnosis. With validation of the nomogram, the model’s discrimination performed better than that predicted by cp-score, stage or age separately. The comprehensive model also showed better calibration. All these proved that at the time of diagnosis, the comprehensive prognosis prediction model can help distinguish patients with different prognosis, and identify those in need of more active treatment and focused monitoring. The nomogram can be considered as a reference tool to formulate clinical treatment and monitoring plans at the time of diagnosis. At the same time, TMs should be reviewed regularly in conjunction with the plan to dynamically adjust the treatment effect and prognosis.

There are some limitations in this research. Although multiple sets of independent samples were used, this is still a single-center study. Differences between institutions, particularly in the context of standardization of pre-analytical and analytical steps, are critical elements that affect the result of biomarker testing. In addition, instead of a randomized controlled trial, this study is a retrospective one that might contain a certain selection bias. Prospective multi-center studies can be carried out in the future to explore the role of TMs in the diagnosis and prognosis of LC.

## Conclusion

In summary, this study established a diagnostic prediction model including CEA, CYFRAR21-1 and NSE, which assists identification of high-risk population for LC more accurately compared with the model considering TMs individually. Moreover, the prognostic prediction model established based on TMs, age and stage can provide an important reference for the development of targeted treatment and monitoring plans for patients with confirmed LC.

## Supplementary Information

**Additional file 1.**

**Additional file 2.**

**Additional file 3.**

## Data Availability

Data supporting the findings of this study are available from the corresponding author upon reasonable request. The datasets generated or analyzed during the study are not publicly available but can be provided from the corresponding author on reasonable request.

## References

[1] Sadate A, Occean BV, Beregi JP, Hamard A, Addala T, de Forges H, Fabbro-Peray P, Frandon J (2020). Systematic review and meta-analysis on the impact of lung cancer screening by low-dose computed tomography. Eur J Cancer.

[2] Bray F, Ferlay J, Soerjomataram I, Siegel RL, Torre LA, Jemal A (2018). Global cancer statistics 2018: GLOBOCAN estimates of incidence and mortality worldwide for 36 cancers in 185 countries. CA Cancer J Clin.

[3] Bade BC, Dela Cruz CS (2020). Lung cancer 2020: epidemiology, etiology, and prevention. Clin Chest Med.

[4] Poggiana C, Rossi E, Zamarchi R (2020). Possible role of circulating tumor cells in early detection of lung cancer. J Thorac Dis.

[5] Joanna K (2018). Arutha, Kulasinghe, Majid E, Warkiani, Ian, Vela, Liz, Kenny: The Prognostic Role of Circulating Tumor Cells (CTCs) in Lung Cancer. Front Oncol.

[6] Knight SB, Crosbie PA, Balata H, Chudziak J, Hussell T, Dive C (2017). Progress and prospects of early detection in lung cancer. OPEN BIOL.

[7] Aberle DR, Adams AM, Berg CD, Black WC, Clapp JD, Fagerstrom RM, Gareen IF, Gatsonis C, Marcus PM, Sicks JD (2011). Reduced lung-cancer mortality with low-dose computed tomographic screening. N Engl J Med.

[8] Shen H (2018). Low-dose CT for lung cancer screening: opportunities and challenges. Front Med.

[9] Gohagan J, Marcus P, Fagerstrom R, Pinsky P, Kramer B, Prorok P (2004). Baseline findings of a randomized feasibility trial of lung cancer screening with spiral CT scan vs chest radiograph: the Lung Screening Study of the National Cancer Institute. Chest.

[10] Becker N, Motsch E, Gross ML, Eigentopf A, Heussel CP, Dienemann H, Schnabel PA, Eichinger M, Optazaite DE, Puderbach M (2015). Randomized study on early detection of lung cancer with MSCT in Germany: results of the first 3 years of follow-up after randomization. J Thorac Oncol.

[11] Sands J, Tammemägi MC, Couraud S, Baldwin DR, Borondy-Kitts A, Yankelevitz D, Lewis J, Grannis F, Kauczor HU, von Stackelberg O (2020). Lung screening benefits and challenges: a review of the data and outline for implementation. J Thorac Oncol.

[12] Fedewa SA, Kazerooni EA, Studts JL, Smith R, Bandi P, Sauer AG, Cotter M, Sineshaw HM, Jemal A, Silvestri GA. State Variation in Low-Dose CT Scanning for Lung Cancer Screening in the United States. J Natl Cancer Inst. 2021;113(8):1044-52. 10.1093/jnci/djaa170 PMC8328984 33176362

[13] Jiang ZF, Wang M, Xu JL (2018). Thymidine kinase 1 combined with CEA, CYFRA21-1 and NSE improved its diagnostic value for lung cancer. Life Sci.

[14] Viani GA, Gouveia AG, Yan M, Matsuura FK, Moraes FY (2022). Stereotactic body radiotherapy versus surgery for early-stage non-small cell lung cancer: an updated meta-analysis involving 29,511 patients included in comparative studies. J Bras Pneumol.

[15] Heagerty PJ, Lumley T, Pepe MS (2000). Time-dependent ROC curves for censored survival data and a diagnostic marker. Biometrics.

[16] Okamura K, Takayama K, Izumi M, Harada T, Furuyama K, Nakanishi Y (2013). Diagnostic value of CEA and CYFRA 21–1 tumor markers in primary lung cancer. Lung Cancer.

[17] Henschke CI, Yankelevitz DF, Libby DM, Pasmantier MW, Smith JP, Miettinen OS (2006). Survival of patients with stage I lung cancer detected on CT screening. N Engl J Med.

[18] Rustad P, Simonsson P, Felding P, Pedersen M (2004). Nordic Reference Interval Project Bio-bank and Database (NOBIDA): a source for future estimation and retrospective evaluation of reference intervals. Scand J Clin Lab Invest.

[19] Rustad P, Felding P, Lahti A (2004). Proposal for guidelines to establish common biological reference intervals in large geographical areas for biochemical quantities measured frequently in serum and plasma. CLIN CHEM LAB MED.

[20] Bjerner J, Høgetveit A, Wold AK, Vangsnes K, Paus E, Bjøro T, Børmer OP, Nustad K (2008). Reference intervals for carcinoembryonic antigen (CEA), CA125, MUC1, Alfa-foeto-protein (AFP), neuron-specific enolase (NSE) and CA19.9 from the NORIP study. Scand J Clin Lab Invest.

[21] Hao C, Zhang G, Zhang L (2019). Serum CEA levels in 49 different types of cancer and noncancer diseases. Prog Mol Biol Transl Sci.

[22] Barouchos N, Papazafiropoulou A, Iacovidou N, Vrachnis N, Barouchos N, Armeniakou E, Dionyssopoulou V, Mathioudakis AG, Christopoulou E, Koltsida S (2015). Comparison of tumor markers and inflammatory biomarkers in chronic obstructive pulmonary disease (COPD) exacerbations. Scand J Clin Lab Invest.

[23] Molina R, Marrades RM, Auge JM, Escudero JM, Vinolas N, Reguart N, Ramirez J, Filella X, Molins L, Agusti A (2016). Assessment of a Combined Panel of Six Serum Tumor Markers for Lung Cancer. Am J Respir Crit Care Med.

