# Supplementary material for: Development and validation of reassigned CEA, CYFRA21-1 and NSE-based models for lung cancer diagnosis and prognosis prediction
Source: BMC Cancer. 2022 Jun 22;22:686. doi: 10.1186/s12885-022-09728-5 (PMC9214980; doi:10.1186/s12885-022-09728-5)
Supplement: Supplementary file 1 — Additional file 1. [file 12885_2022_9728_MOESM1_ESM.pdf]

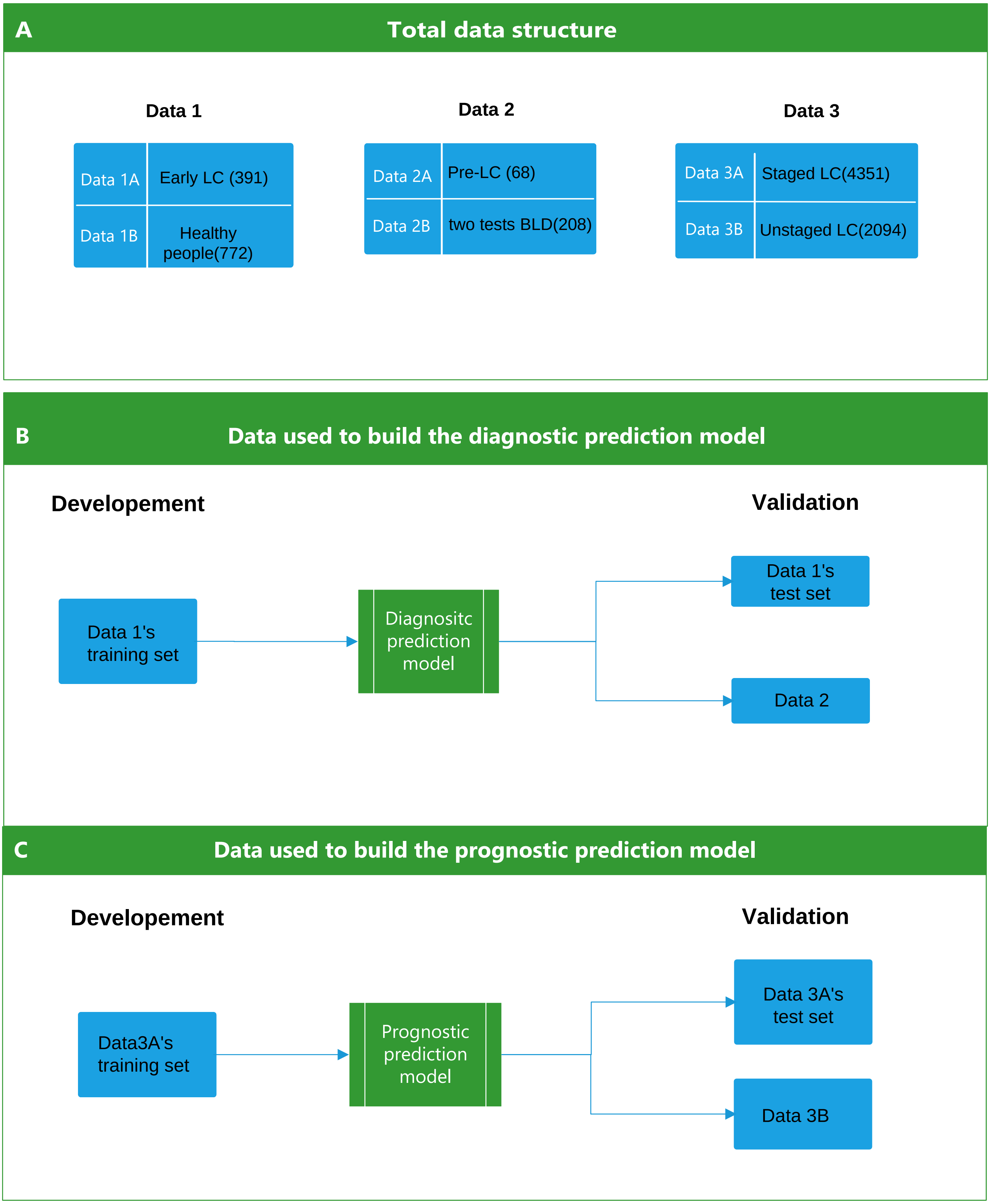

Supplementary figure 1. Data structure of development and validation for diagnostic prediction model and prognostic prediction model. **Data 1**,Early LC patients and healthy people meet the criteria for LC screening; **Data 2A**, LC patients who had tumor marker tests at least 1 month prior to diagnosis; **Data 2B,** BLD patients who had two tumor marker tests at least 1 month apart; **Data 3A,** LC patients with defined stage; **Data 3B**, LC patients whose tumor stage is not clearly defined. LC, lung cancer. BLD, benign lung disease.

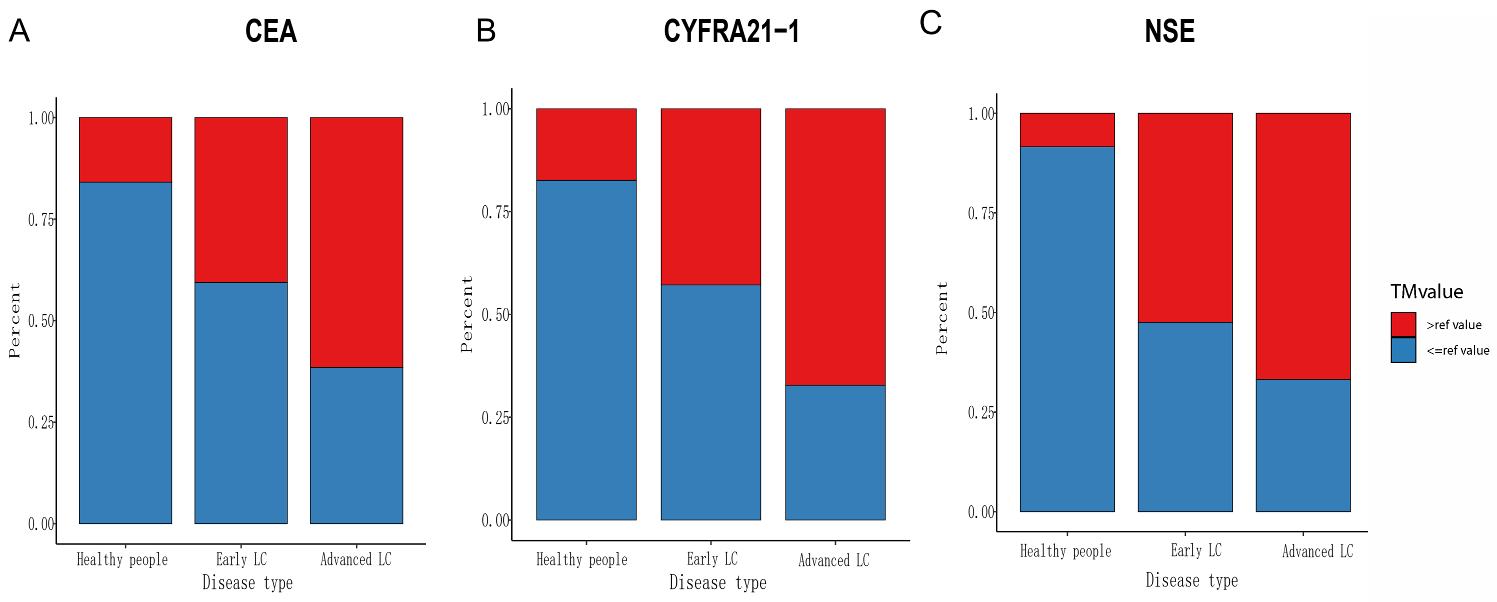

Supplementary figure 2. The proportion of CEA(A), CYFRA21-1(B) and NSE(C) in different population that are higher than the reference value.

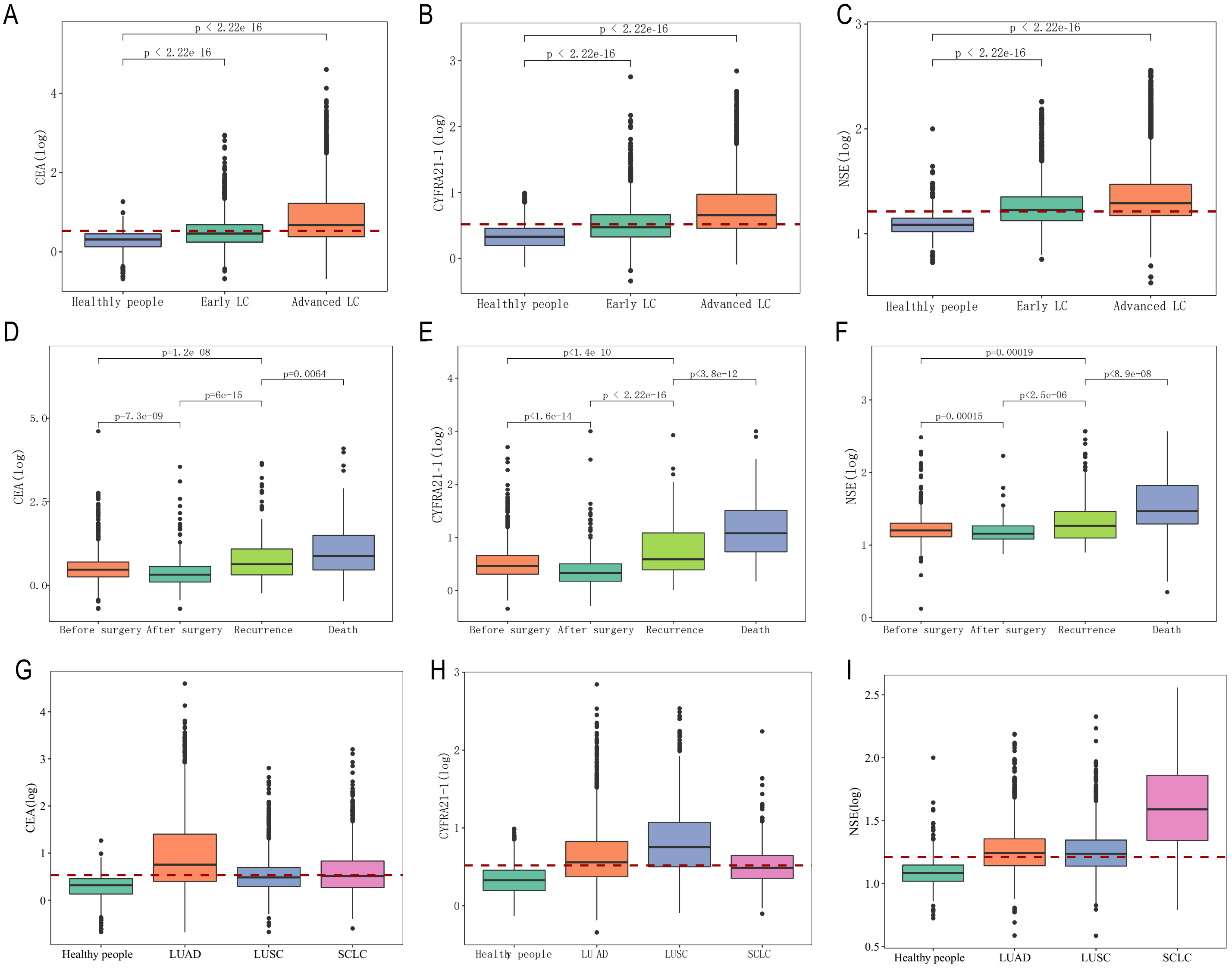

Supplementary figure 3. The distribution of CEA, CYFRA21-1 and NSE values after logarithmic transformation in different population(A-C), treatment period (D-F), and histological types(G-I). LC, lung cancer. LUAD,Lung adenocarcinoma. LUSC,Lung squamous cell carcinoma.SCLC,Small Cell Lung Cancer.

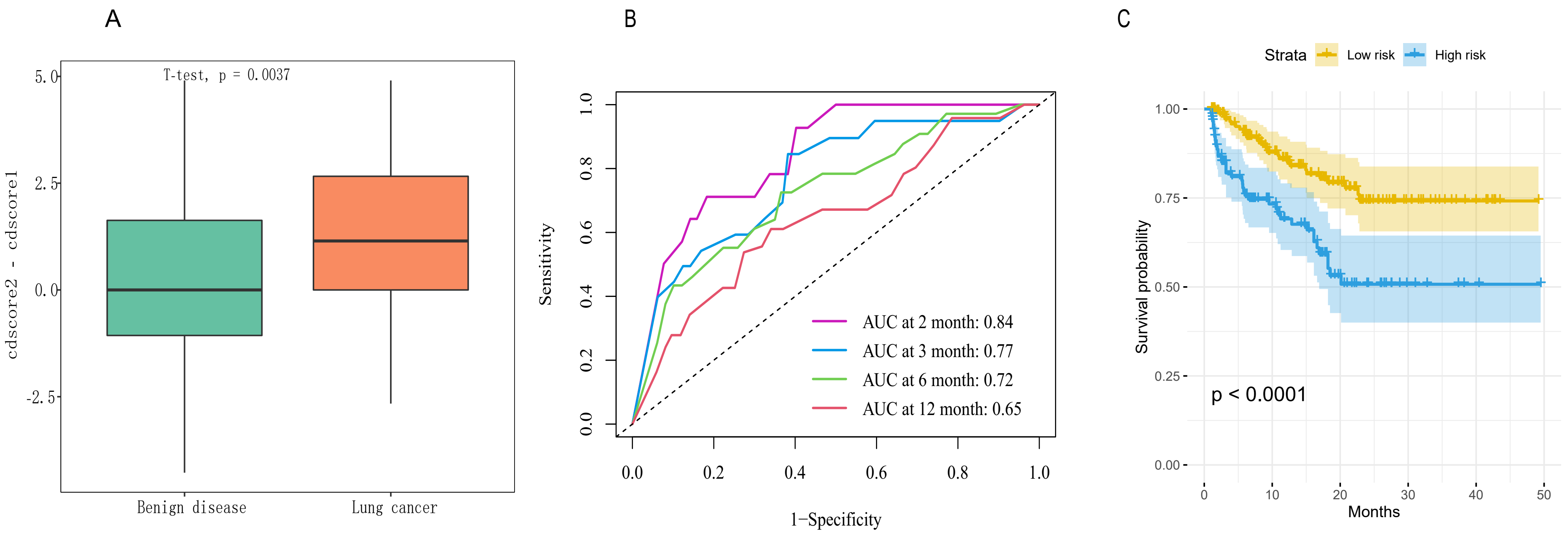

Supplementary figure 4. Validation of the ability of cd-score to predict the risk of lung cancer diagnosis. (A) The difference between the two measurements of cd-score in lung cancer and benign patients. (B) Time-dependent ROC and corresponding AUCs for 2-, 3-, 6-,12-month LC diagnosis predicted by cd-score1. (C) Survival curves of benign patients with low or high risk of LC diagnosis according to the cd-score1.

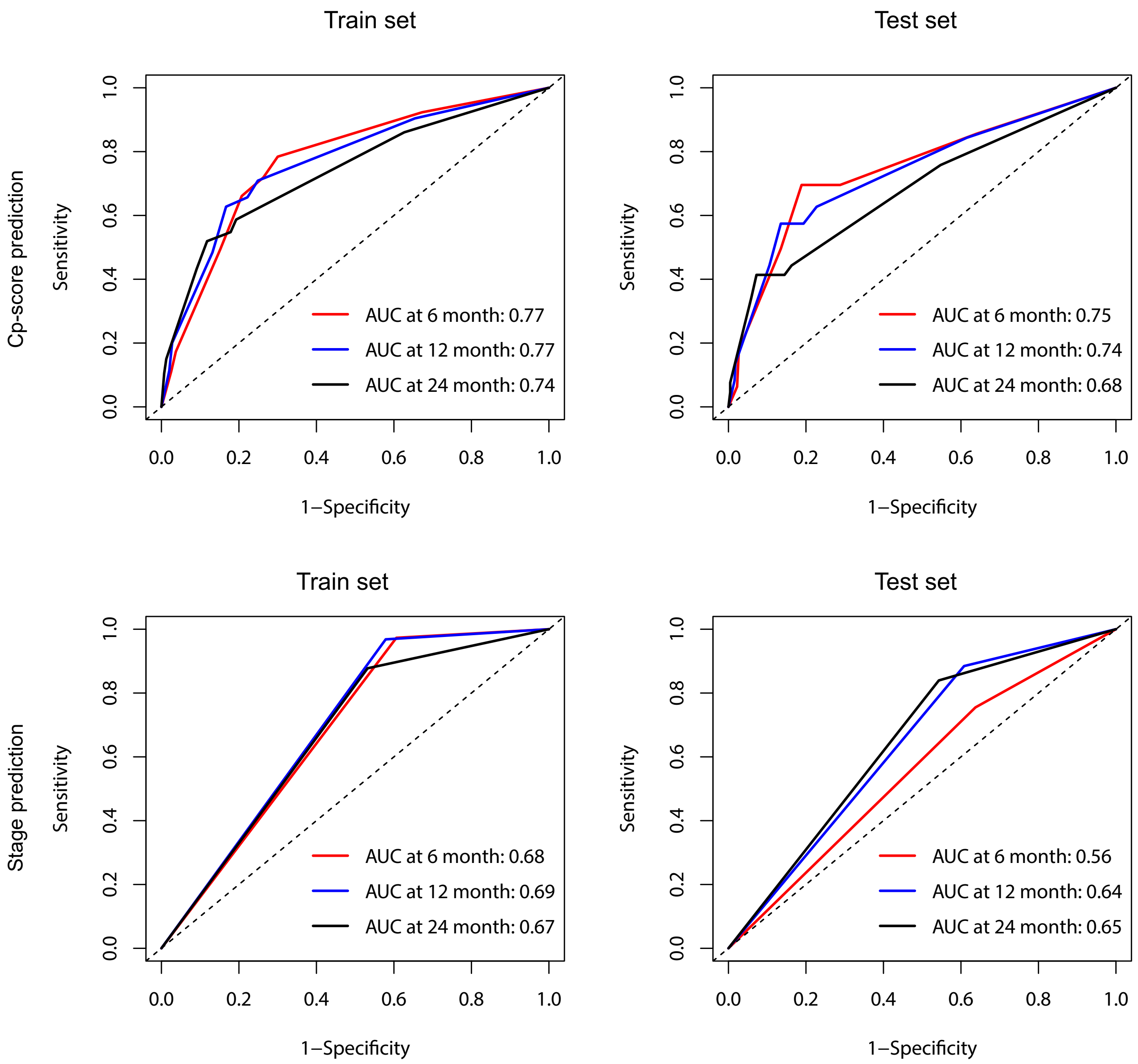

Supplementary figure 5. Time-dependent ROC and corresponding AUCs for 6-,12-,24month survival predicted by cp-score and stage in training set and test set.

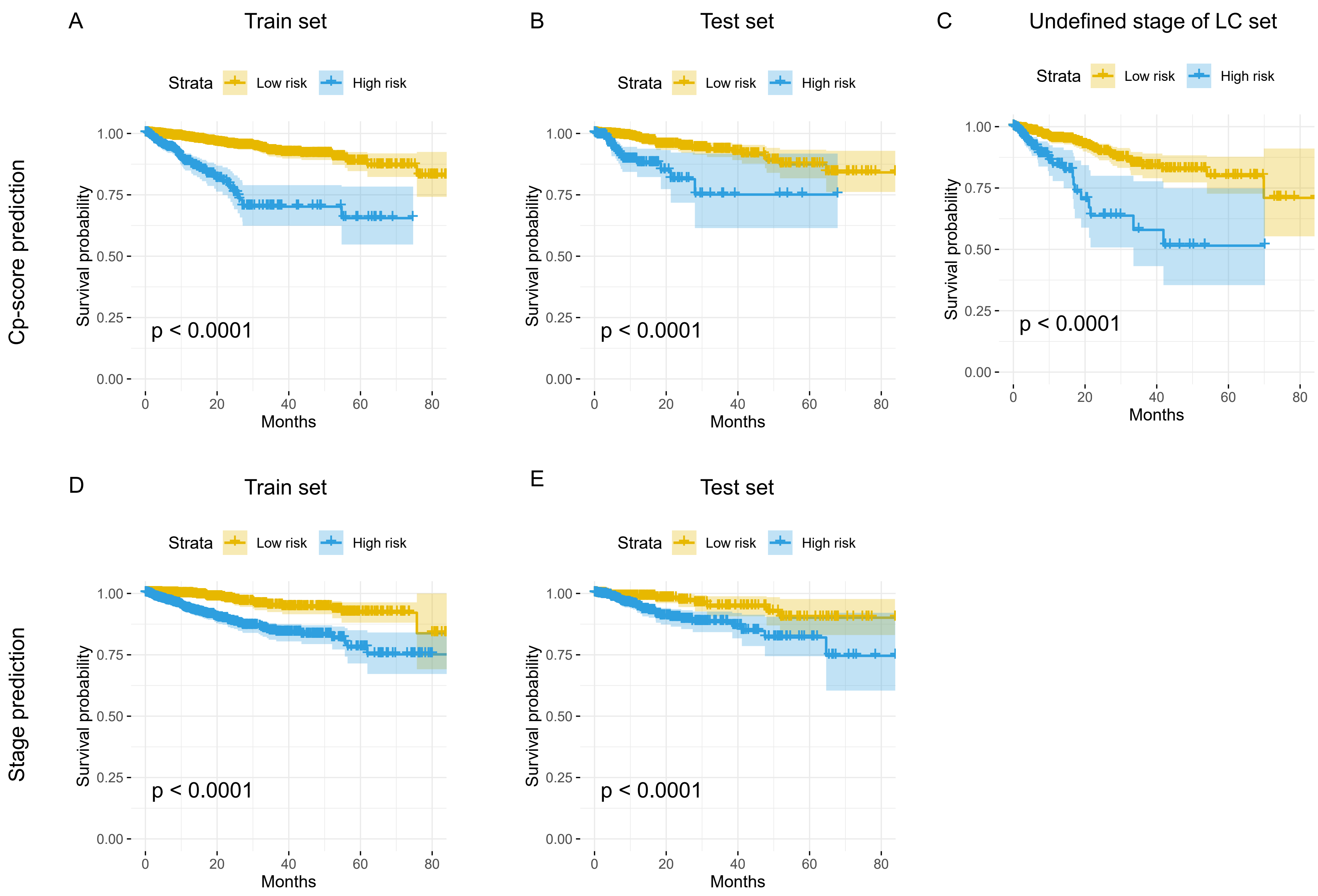

Supplementary figure 6. Survival curves of lung cancer patients with low or high risk of death according to cp-score in training set (A), test set (B) and Data 3A (C) and stage in training set (D) and test set(E).
