# Supplementary material for: Development and validation of reassigned CEA, CYFRA21-1 and NSE-based models for lung cancer diagnosis and prognosis prediction
Source: BMC Cancer. 2022 Jun 22;22:686. doi: 10.1186/s12885-022-09728-5 (PMC9214980; doi:10.1186/s12885-022-09728-5)
Supplement: Supplementary file 2 — Additional file 2. [file 12885_2022_9728_MOESM2_ESM.docx]

**Supplementary Materials and Methods**

TM records of LC patients at diagnosis were defined as the test taken between 14 days before to 5 days after the diagnosis. For more than one test taken, the test most recent to diagnosis would be selected. The TNM staging of NSCLC was determined based on the 7^th^ edition of *The American Joint Committee on Cancer* (AJCC), and the 2-stage system proposed by a Veterans Lung Study Group (VALG) in 1973. In this study, NSCLC stage I, stage II, and SCLC limited stage were collectively referred to as early stage; and NSCLC stage III, IV, and SCLC expansion stage as advanced stage. All patients with lung cancer were followed up since diagnosis, and the endpoint event was set as death. The follow-up period ended in June, 2020.

**Development and validation of the diagnostic model**

Data 1 was randomly divided into a training data set and a test data set at a ratio of 7:3, which was used to build and validate the model respectively. Some extreme TM values might lead to the instability of the established model, but deletion of these values might result in loss of effective information. Therefore, based on TM distribution in Data 1’s training data set, TM was re-assigned with two cut-off values to become five classification variables. The first cutoff value was the 10% quartile of TM in patients with early-stage LC. The second cutoff value was the best threshold according to Youden-Index from the receiver operating characteristic (ROC) curve, in which the independent and dependent variables were single TM value and disease diagnosis in LC patients and healthy people, respectively. Name reassigned TM variables as CEAmod, CYFRA 21-1mod and NSEmod (supplementary Table 2).

Taking three newly assigned TMs as independent variables and the diagnosis of disease as the dependent variable, logistic regression was performed to establish a diagnostic prediction model, and the resulting comprehensive prediction score was named cd-score. Compare the model with prediction by each individual TM. Models were validated by test sets of Data 1 and Data 2. The ROC curve was used to evaluate the discrimination while calibration chart and DCA curve were used to evaluate the calibration and clinical validity. A nomogram was used to present the diagnosis prediction model.

To provide better guidance on screening of high-risk LC population, we introduced quantified value to predict their risk of LC in future. We calculated cd-score corresponding to the patient’s first and last TM test in Data 2A and Data 2B, which were recorded as cd-score 1 and cd-score 2, respectively. The time-dependent ROC curve and Kaplan-Meier curves were used to evaluate the discrimination ability of the cd-score.

**Development and validation of the predictive model for prognosis and survival**

We followed up patients with confirmed LC. Death was set to be the endpoint event. LC patient data (Data 1A) was randomly divided into a training set and a test set at a ratio of 7:3. In the training data set, a time-dependent ROC curve was drawn to demonstrate 12-month survival predicted by each individual TM. Based on Youden-index, we got the optimal threshold to re-assign TMs in the training data set and the test data set, turning them into binary variables, namely CEAp, CYFRA21-1p and NSEp. Include three re-assigned TMs into the COX regression model, from which their ability to predict LC survival was analyzed, and a comprehensive prognostic prediction score (cp-score) was obtained. By the same method, we established a stage-based prognostic prediction model to compare its prediction capability with cp-score. In the end, in order to provide effective guidance on clinical practice, we brought CEAp, CYFRA21-1p, NSEp, age and stage into the COX regression model at the same time, from which a comprehensive prognostic prediction model was established. The model was presented with a nomogram, showing the survival rate of LC patients in 1, 2, and 3 years after the diagnosis. The time-dependent ROC curve and Kaplan-Meier curves were used to evaluate the discrimination ability of the integrated model, and the calibration chart was used to evaluate the calibration.
