# Supplementary material for: Development and validation of reassigned CEA, CYFRA21-1 and NSE-based models for lung cancer diagnosis and prognosis prediction
Source: BMC Cancer. 2022 Jun 22;22:686. doi: 10.1186/s12885-022-09728-5 (PMC9214980; doi:10.1186/s12885-022-09728-5)
Supplement: Supplementary file 3 — Additional file 3. [file 12885_2022_9728_MOESM3_ESM.docx]

Supplementary Table **1 Clinical characteristics of the entire study cohort**

| **Characteristic** | **Early LC** | **Healthy people** | **Pre-LC** | **BLD (test>2)** | **LC (Staged)** | **LC (Unstaged)** |
| --- | --- | --- | --- | --- | --- | --- |
| **Data code** | Data 1A | Data 1B | Data 2A | Data 2B | Data 3A | Data 3B |
| **Total NO.** | 391 | 772 | 68 | 208 | 4351 | 2094 |
| **Age (mean[SD])** | 60.63(8.14) | 58.76(6.96) | 66.22(10.44) | 68.51(12.90) | 61.19(9.84) | 64.55(10.24) |
| **Sex -No. (%)** |  |  |  |  |  |  |
| Female | 168(43.0) | 219(28.4) | 18(26.5) | 64 (30.8) | 1290(29.6) | 612(29.2) |
| Male | 223(57.0) | 553(72.6) | 50(73.5) | 144 (69.2) | 3061(70.4) | 1482(70.8) |
| **Stage-no.(%)** |  |  |  |  |  |  |
| Early | 391(100.0) | - | 25(36.76) | - | 1415(32.5) | - |
| Advanced | - | - | 19(27.94) | - | 2936(67.5) | - |
| Unstage | - | - | 24(35.3) | - | - | 2094(100.0) |
| **Pathological type-no.(%)** | |  |  | - |  |  |
| NSCLC | 304(77.7) | - | 62(91.17) | - | 3618(83.1) | 1883(89.9) |
| SCLC | 87(22.3) | - | 8(11.76) | - | 733(16.9) | 211(10.1) |
| **TM when diagnosed ng/ml(median[IQR])** | | |  |  |  |  |
| CEA | 2.84 (3.55) | 2.11 (1.48) | 4.05(5.81) | 2.41(2.33) | 3.81(8.09) | 3.95(7.99) |
| CYFRA21-1 | 3 (2.19) | 2.16 (1.33) | 4.195(5.74) | 2.35(1.47) | 3.91(5.07) | 4.23(6.29) |
| NSE | 17.78 (9.21) | 12.16 (3.59) | 18.14(9.51) | 14.645(7.56) | 18.5(12.16) | 17.91(11.37) |
| **First TM test in Data 2(median[IQR])** | | |  |  |  |  |
| CEA | - | - | 3.37(4.65) | 2.3(2.07) | - | - |
| CYFRA21-1 | - | - | 2.99(2.97) | 2.44(1.65) | - | - |
| NSE | - | - | 14.23(5.81) | 13.1(6.34) | - | - |
| **Median time between two tests(month)** | - | - | 5.9 | 14.25 | - | - |

**Abbreviations:** LC, lung cancer; BLD, lung benign disease; BD, benign disease. TM, tumor marker;

Supplementary Table 2. The threshold values used in TM re-assignment

| In diagnostic model | Cutoff 1 | Cutoff 2 |
| --- | --- | --- |
| CEAmod | 1.46 | 3.33 |
| CYFRA21-1mod | 1.94 | 3.04 |
| NSEmod | 11.29 | 14.73 |
| In prognostic model | Cutoff 1 |  |
| CEAp | 3.12 | - |
| CYFRA21-1p | 33.64 | - |
| NSEp | 6.94 | - |

Supplementary Table 3. Characteristics of the three markers and their coefficients in prognosis.

|  | **Coefficients** | **HR** | **CI (lower)** | **CI (upper)** | **z value** | **p value** |
| --- | --- | --- | --- | --- | --- | --- |
| Age | 0.04 | 1.04 | 1.02 | 1.07 | 4.32 | <0.001 |
| CEAp>3.12 | 0.59 | 1.8 | 1.21 | 2.69 | 2.87 | 0.004 |
| NSEp>33.64 | 1.08 | 2.95 | 1.97 | 4.42 | 5.23 | <0.001 |
| CYFRA21-1p>6.94 | 0.84 | 2.33 | 1.61 | 3.35 | 4.52 | <0.001 |
| Advanced | 1.15 | 3.16 | 1.91 | 5.22 | 4.49 | <0.001 |
